# Supplementary material for: Content-rich biological network constructed by mining PubMed abstracts
Source: BMC Bioinformatics. 2004 Oct 8;5:147. doi: 10.1186/1471-2105-5-147 (PMC528731; doi:10.1186/1471-2105-5-147)
Supplement: Additional File 5 — The original Chilibot query results of the term "long-term potentiation (LTP)" and 22 other terms, limiting the latest references analyzed to the years 1990, 1995, 2000, and 2004. [file 1471-2105-5-147-S5.bz2 › chilibotAdditionalFile5/ltp2000/html/LTP_ARC.html]

 


 **LTP** and **ARC** 
  
Found 1 abstracts in PubMed,  **1 abstracts were retrieved and analyzed**.  


---

 Search Google  |
 PDF files only 
|  EDU domain only 

---

**Interactive relationship** (e.g. stimulation, inhibition, etc)

- Inhibition of activity dependent  **arc**  protein expression in the rat hippocampus impairs the maintenance of long term potentiation  [ **LTP** ]  and the consolidation of long term memory.  Ref: 10818134 J Neurosci, 2000
- Our studies show that disruption of  **Arc**  protein expression impairs the maintenance phase of  **LTP**  without affecting its induction and impairs consolidation of LTM for spatial water task training without affecting task acquisition or short term memory.  Ref: 10818134 J Neurosci, 2000

- :-)

  **Parallel relationship** (e.g. studied together, co-existance, homology, etc.)

  - We used intrahippocampal infusions of antisense oligodeoxynucleotides to inhibit  **Arc**  protein expression and examined the effect of this treatment on both  **LTP**  and spatial learning.  Ref: 10818134 J Neurosci, 2000
